# Advancing the Science and Application of Learning Health Systems in General Medical and Primary Care Practice

**DOI:** 10.1007/s11606-026-10676-0

**Published:** 2026-08-10

**Authors:** Donna L. Washington, Shari Bolen, Jeremy B. Sussman

**Affiliations:** 1 VA HSR Center for the Study of Healthcare Innovation, Implementation, & Policy, VA Greater Los Angeles Healthcare System, Los Angeles, CA USA; 2 Department of Medicine, David Geffen School of Medicine, University of California, Los Angeles, Los Angeles, CA USA; 3 Department of Medicine, Case Western Reserve University at The MetroHealth System, Cleveland, OH USA; 4 Department of Population and Quantitative Health Sciences, Case Western Reserve University at The MetroHealth System, Cleveland, OH USA; 5 VA Center for Clinical Management Research, VA Ann Arbor Healthcare System, Ann Arbor, MI USA; 6 Department of Medicine, University of Michigan, Ann Arbor, MI USA

Learning health care systems were first conceptualized by the Institute of Medicine (now National Academy of Medicine) as “systems in which science, informatics, incentives, and culture are aligned for continuous improvement and innovation, with best practices seamlessly embedded in the care process, patients and families active participants in all elements, and new knowledge captured as an integral by-product of the care experience.”^1^ The imperative for development of learning health systems were the gaps in all realms of US health care quality and the evidence for the origin of these gaps. These gaps were driven in part by variation in adoption of new scientific evidence and innovations; health system infrastructures that were not designed to collect, interpret, and act on real-world evidence from their clinical practices; incentive systems not aligned with team-based, patient-centered, high value care; and absence of a culture explicitly focused on continuous learning and quality improvement.^1^ Since its early conceptualization, the frameworks, science, adoption, and implementation strategies for learning health systems have substantially developed. Despite this progress, the imperative for learning health systems remains. US health care systems remain challenged to deliver on the vision for high quality, high-value, patient-centered care that incorporates innovations in therapeutics and information systems while mitigating quality, safety, fiscal, and other challenges.

The foundation for a learning health system is continuous knowledge development, improvement, and application.^1^ This is illustrated in the three-phase learning health system learning cycle, in which clinical practice is captured as data (performance-to-data phase), data are assembled and analyzed and integrated with the scientific evidence toward greater understanding (data-to-knowledge-generation phase), and this is used to inform interventions to improve practice change (knowledge-to-performance phase) in continuous, replicating learning cycles.^2^ Perhaps the most widespread illustration of a framework for implementing this process is the Quality Enhancement Research Initiative (QUERI) Implementation Roadmap developed within the U.S. Department of Veterans Affairs (VA)—the largest integrated health care system and learning health care system in the USA.^3^ QUERI aims to accelerate the adoption of evidence-based practices into routine practice in VA.^3^ The QUERI Implementation Roadmap aligns with the learning health system by systematically guiding identification, implementation, and sustainment of evidence-based practices within VA, e.g., through guiding prioritization and engagement of key interested groups and clinical operations leadership, evaluation processes, and technical support. In addition, the QUERI Implementation Roadmap may be used to characterize future directions in the research and application of learning health systems.

The collection herein of the *Journal of General Internal Medicine* offers original research and perspectives on advancing the science and application of learning health care systems in general medical and primary care practice. These studies describe learning health systems across a variety of settings and clinical content areas that illustrate the promise as well as the challenges of learning health system design, implementation, and research. Settings for the implementation of learning health systems in this collection include a large federally qualified health center (FQHC) serving over 110,000 outpatients annually and providing family support services in a community-based setting, a large academic medical center serving as a hub for 10 clinical sites for hypertension care, and distinct virtual, rural, research network, multisite, and program-based settings or programs within the national VA health care system.^4–13^ Clinical trial number not applicable.

The range of general medical and primary care settings in this collection across which learning health systems were implemented highlights the commonalities in learning health system implementation and strategies. Crowley and colleagues applied the Learning Health System Consolidated Framework (LHS-CF) taxonomy to examine real-world practice change of a multisite telehealth program within VA for patients with poorly controlled diabetes.^4^ They noted that the LHS-CF explicitly defines enabling conditions that facilitate learning health system work and bodies of work that promote improved patient care within learning health systems.^4^ Their learning health system program was associated with an improvement in diabetes control at 6 months that was sustained at 4 years. Gore and colleagues used learning health system strategies to align research with clinical priorities, and support clinical partners, data analysis and dissemination of findings in their FQHC learning health system, and applied the Consolidated Framework for Implementation Research (CFIR) to identify implementation barriers and facilitators.^5^ They described the challenges and their strategies for integrating research with health care delivery in their FQHC setting, which was essential for building a learning health system. They described supportive leadership, a preexisting culture of continual quality improvement, academic partners, and a modular and iterative learning health system development approach as key strategies for enabling FQHCs to integrate research with health care delivery in safety-net primary care.^5^

Addressing the role of leadership and of partnerships in building a learning health system were themes that were also addressed in studies by Bravata^8^ and by Sides.^9^ Bravata evaluated discrete strategies that geriatric service leadership deployed to construct a VA geriatrics learning health system to support older Veterans transitioning from hospital to home. That study identified specific leadership strategies that fostered stakeholder participation and informed data-driven improvements, stating their geriatric learning health system “illustrates the importance of active listening, collaborative engagement, and responsive adaptation to advance objectives.”^8^ Sides, by contrast, evaluated patient engagement activities and infrastructure across seven VA health systems research networks.^9^ That evaluation was framed by the Patient-Centered Outcomes Research Institute’s National Patient-Centered Clinical Research Network framework. They identified discrete engagement-related activities within three domains—facilitating patient-engaged research, network engagement infrastructure, and building and maintaining relationships with partners.^9^ Though their findings identified important considerations for patient engagement, they also highlighted a need for future research to understand how their findings vary within different contexts or health care settings.

Digital capture of the care experience is an important component of learning health systems that represents an emerging area of inquiry for novel data sources. The study by Mak investigated the perspectives of clinical care providers and holistic/wellness providers on integrating consumer-grade wearable devices and patient-generated health data into clinical care.^10^ This is an example of the type of pre-implementation activities described in the QUERI Implementation Roadmap. Participants in this study expressed perceived value from integration of novel data into workflow and patient interactions, but they also identified infrastructure, privacy, and educational needs that required addressing in advance of implementation.

Leveraging learning health systems and implementation science to address health equity is the subject of two of the perspectives in this collection.^11, 12^ Advancing health equity, where everyone would have a fair and just opportunity to attain their highest level of health, is one of the quintuple aims of learning health systems. Shannon describes a research and clinical operations partnership to improve health outcomes, quality, and patient experience across the VA.^11^ The toolkit developed through that partnership combines data analytics with actionable strategies to address unwarranted gaps in care. Wong describes two case examples, also within the VA, that incorporate an equity focus into implementation strategies for evidence-based practices to improve care.^12^ In those case examples, the equity lens was applied to the implementation of existing evidence-based practices. Together, these perspectives illustrate approaches for leveraging learning health systems in pursuit of improvements in population health and equity. A third perspective looks retrospectively at learning health system models implemented during the COVID-19 pandemic, to highlight challenges to maintaining learning health systems.^13^

These papers show that we are early in the development of the learning health system, but we are also at a point that further development can occur. A meaningful learning health system needs a certain maturity of specific infrastructures related to payment systems, technology, partner engagement (e.g., patient, family, and community), and quality improvement, which is even now only occasionally possible. This collection on learning health systems in general medical and primary care practice is meant to offer new insights and perspectives, and also to spur future health system innovations and scientific inquiry. These papers demonstrate several areas ripe for future learning health system research: (1) pre-implementation and sustainability of learning health systems; (2) understanding ways to improve structural and informatics aspects of learning health systems such as how to incorporate artificial intelligence tools; (3) education or training related to learning health systems; and (4) integration of patient, community, and payer engagement and collaboration. Collectively, this work reveals the promise and challenges of learning health systems.

## Data Availability

Not applicable.
